# Chemical Induction of Trophoblast Hypoxia by Cobalt Chloride Leads to Increased Expression of DDIT3

**DOI:** 10.1134/S1607672921040104

**Published:** 2021-08-23

**Authors:** E. N. Knyazev, S. Yu. Paul, A. G. Tonevitsky

**Affiliations:** 1 Shemyakin–Ovchinnikov Institute of Bioorganic Chemistry, Russian Academy of Sciences, Moscow, Russia; 2 Faculty of Biology and Biotechnology, National Research University Higher School of Economics, Moscow, Russia; 3 Translational Technology Center, Moscow, Russia; 4 Troitsk Research and Development Center, Moscow, Russia

**Keywords:** placenta, choriocarcinoma, BeWo, hypoxia, cobalt, hydroxyquinoline, unfolded protein response, DDIT3, CHOP, mTORC1

## Abstract

Choriocarcinoma cells BeWo b30 are used to model human placental trophoblast hypoxia using cobalt (II) chloride and hydroxyquinoline derivative (HD) as chemical inducers of hypoxia-inducible factor (HIF). In this study, it was shown that both substances activate the hypoxic pathway and the epithelial–mesenchymal transition and inhibit the pathways of cell proliferation. However, CoCl_2_ caused activation of the apoptosis pathway, increased the activity of effector caspases 3 and 7, and increased the expression of the unfolded protein response target DDIT3. The mTORC1 pathway was activated upon exposition to CoCl_2_, while HD suppressed this pathway, as it happens during real trophoblast hypoxia. Thus, effect of CoCl_2_ on BeWo cells can be a model of severe hypoxia with activation of apoptosis, while HD mimics moderate hypoxia.

During normal development of the placenta, on days 9–10 day after fertilization, cytotrophoblast cells migrate into the uterine spiral arteries and replace the endothelium, forming vessels with a high flow, which are resistant to vascular tone regulators. Disturbance of the trophoblast invasion into the vessels due to hypoxia and other factors leads to impaired blood supply, endoplasmic stress, and increased apoptosis of trophoblast cells [1].

Studies of the biology of the placenta during pregnancy in humans is limited for ethical reasons, and the placenta after normal delivery or caesarean section does not reflect the early stages of placental development, which are important for the pathogenesis of preeclampsia. Animal models of the placenta differ from human models both at the anatomical and molecular levels [2]. In this regard, in vitro models of the placental barrier are of particular importance. Primary human trophoblast cells have a limited division potential and low reproducibility of results when using cells from different donors. The use of immortalized cell lines that mimic normal trophoblast, such as the human choriocarcinoma line BeWo b30, allows for better reproducibility, and the use of the extracellular matrix and microfluidic devices for simulating the natural microenvironment and blood flow brings the in vitro models even closer to physiological conditions [3].

The central factor in the hypoxic pathway activation is the accumulation of hypoxia-inducible factor 1 alpha (HIF-1α). During normoxia, HIF-1α is hydroxylated by HIF-prolyl hydroxylases (PHD), which creates conditions for the attachment of the von Hippel–Lindau protein (pVHL) and triggering ubiquitinylation and proteasome degradation of HIF-1α [4]. Under conditions of hypoxia, PHD activity is suppressed, the level of HIF-1α increases, it is translocated into the nucleus and undergoes heterodimerization with the constitutive subunit HIF-1β, which causes transcription of target genes [5].

Hypoxia induction with CoCl_2_ is achieved by replacing Fe^2+^ ions in the active site of PHD with Co^2+^ ions. CoCl_2_ also induces the oxidation of ascorbates, which are important cofactors of PHD, binds to HIF-1α, thereby preventing the interaction with pVHL, and inhibits asparaginyl hydroxylase FIH, which inhibits HIF. The mechanisms of action of CoCl_2_ are described in more detail in the review [6]. It should be noted that the genes whose expression changes under the influence of CoCl_2_ only partially overlap with those under oxygen deficiency, which can be explained by the effect of CoCl_2_ on other enzymes and signaling pathways in the cell [7] and brings into question the relevance of this model of hypoxia.

It was previously shown that hydroxyquinoline derivatives (HD), which bind the Fe^2+^ ion in the active site of PHD, but not other known enzymes of this family, can also be used for the chemical simulation of hypoxia, which leads to inhibition of HIF-1α hydroxylation, its accumulation in the cell, and activation of the hypoxia pathway [8]. Substituents at position 7 of HD mimic the structure of the HIF-1α domain, which contacts with the active site of PHD. This provides specific binding to these enzymes and reduces the number of side effects as compared to the use of CoCl_2_ [9, 10].

The aim of this study was to compare the changes in cells at the transcriptome level under the influence of CoCl_2_ and HD on BeWo b30 choriocarcinoma cells.

Choriocarcinoma cells BeWo b30 were grown in 6-well plates in DMEM medium with L-glutamine and glucose (4.5 g/L), supplemented with 10% One Shot fetal bovine serum, 1% 100x MEM NEAA, and 1% 100x Pen Strep. After reaching 80% confluence, the cell medium was replaced with a medium containing 300 µM CoCl_2_ or 5 µM HD 4896–3212. Under control conditions, the culture medium was replaced with a fresh one. After 24 h of incubation, we assessed the metabolic activity of cells in the MTT test and the activity of caspases 3 and 7 as an indicator of the effector phase of apoptosis using the Abcam Caspase-3 Assay Kit.

Total RNA was isolated by guanidine thiocyanate–phenol–chloroform extraction [11] using the Qiagen miRNeasy Mini Kit. The RNA concentration was determined using a NanoDrop 1000 instrument [12]. The RNA quality was assessed using a Bio-Rad Experion instrument by the RQI value [13].

Next-generation sequencing was performed on an Illumina NextSeq 500 with the preparation of libraries using the Illumina Stranded mRNA Library Prep Kit. The change in gene expression was considered significant when the multiplicity of differences was more than 2.0 and the *p* value for the Student’s *t* test with the Benjamini–Hochberg correction for the multiplicity of comparisons was less than 0.05 [14]. Activated and suppressed signaling pathways were identified using the MSigDB collection.

Severe hypoxia, oxidative stress induced by reactive oxygen species (ROS), and endoplasmic stress can cause activation of the internal signaling pathway of apoptosis. The effector phase of apoptosis is associated with the activation of caspases 3 and 7, which determine the main processes of apoptotic transformation of cells [15]. The relative activity of caspases 3 and 7 and the metabolic activity according to the MTT test are shown in Table 1. It can be concluded from the data obtained that, under the action of CoCl_2_, the activation of apoptosis in cells is more pronounced, which imitates the more severe hypoxia of the trophoblast as compared with HD. The absence of differences in the results of the MTT test between the two treatments indicates that both substances have practically no significant effect on the activity of cell mitochondria as a response to the hypoxia pathway activation.

**Table 1.** Metabolic activity and apoptosis in BeWo b30 cells

Index	Control	HD	CoCl_2_
Metabolic activity according to MTT test, %	100 ± 5	81 ± 4*	82 ± 5*
Caspase 3 and 7 activity, %	100 ± 17	147 ± 11*	213 ± 11*

* *p* < 0.05.

Incubatation of BeWo b30 cells with CoCl_2_ and HD for 24 h led to a statistically significant more than twofold change in the expression of 3030 and 1030 genes, respectively; the expression of 287 genes changed unidirectionally in both cases. With both types of exposure, the hypoxia pathway and the epithelial–mesenchymal transition were activated, and the activity of the pathways associated with the transcription factors of the E2F family and the G2M checkpoint (i.e., cell proliferation pathways) was inhibited. This coincides with the effects of hypoxia on the placenta in vivo: activation of HIF-1α in trophoblast cells suppresses division and stimulates cell motility, which leads to migration of trophoblast cells and their invasion into uterine vessels [1].

Figure 1a shows the expression profiles of the genes of the hypoxia pathway in the control cell samples and under exposure to HD and CoCl_2_, indicating the activation of the same HIF-1α targets. The analysis of the genes whose expression significantly changed uniquely under the influence of CoCl_2_ revealed the activation of the apoptosis pathway and the response to ROS. This confirms the data that cobalt can induce ROS accumulation, causing DNA damage and leading to apoptosis [16].

**Fig. 1.**
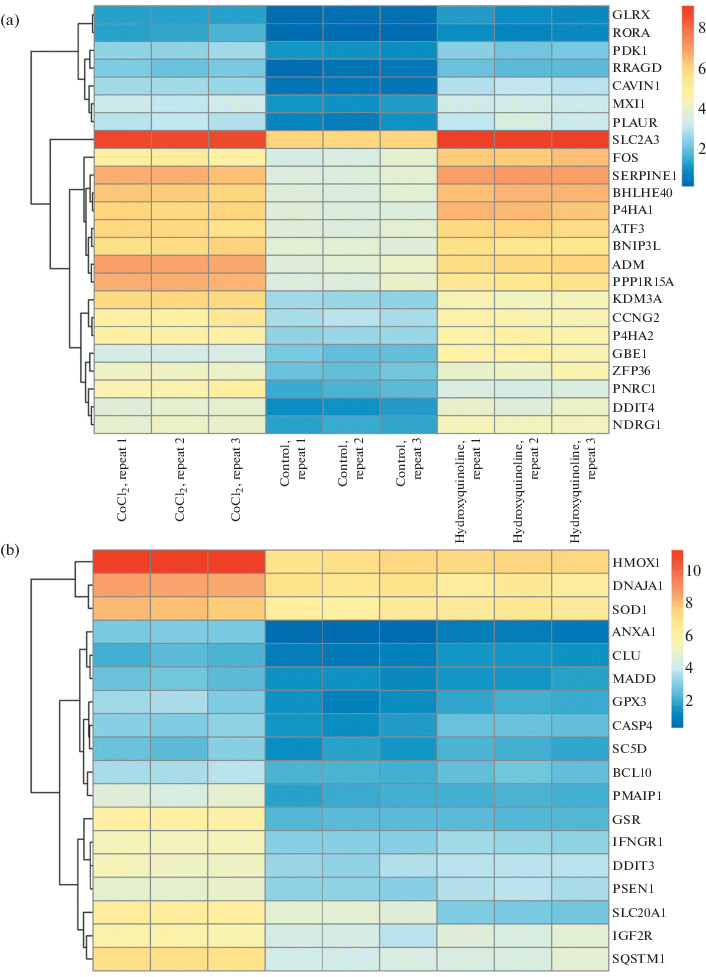
Expression of genes related to the hypoxia signaling pathway (a) and apoptosis (b) in control and upon exposure to HD and CoCl_2_.

Among the genes whose expression changed only upon exposure to HD, activation of these pathways was not observed. Figure 1b shows the expression of genes of the apoptosis pathway, indicating that the picture in the control and under the influence of HD is practically the same, whereas cobalt causes a significant activation of this pathway.

Hypoxia, which is accompanied by the production of ROS and cytoplasmic stress, leads to disruption of proper protein folding and activation of the unfolded protein response. This pathway leads to the adaptation of the cell to hypoxia; however, severe hypoxia, exceeding the compensatory capabilities of the cell, leads through this pathway to the accumulation of the transcription factor CHOP, encoded by the *DDIT3* gene, which triggers apoptosis [17]. Under the influence of HD, the expression of *DDIT3* did not change significantly; however, the incubation with CoCl_2_ increased the expression of *DDIT3* 3.9 times (*p* < 0.001). Figure 2 shows a simplified scheme of the unfolded protein response, and an increase in CHOP expression, found in our study, can be seen. Apparently, the effect of CoCl_2_ mimics a more severe form of hypoxia in comparison with HD. It was shown that another HD, known as adaptachine, prevents the activation of apoptosis through the branch of the response to unfolded proteins PERK/ATF4/CHOP [18], which is consistent with our observations.

**Fig. 2.**
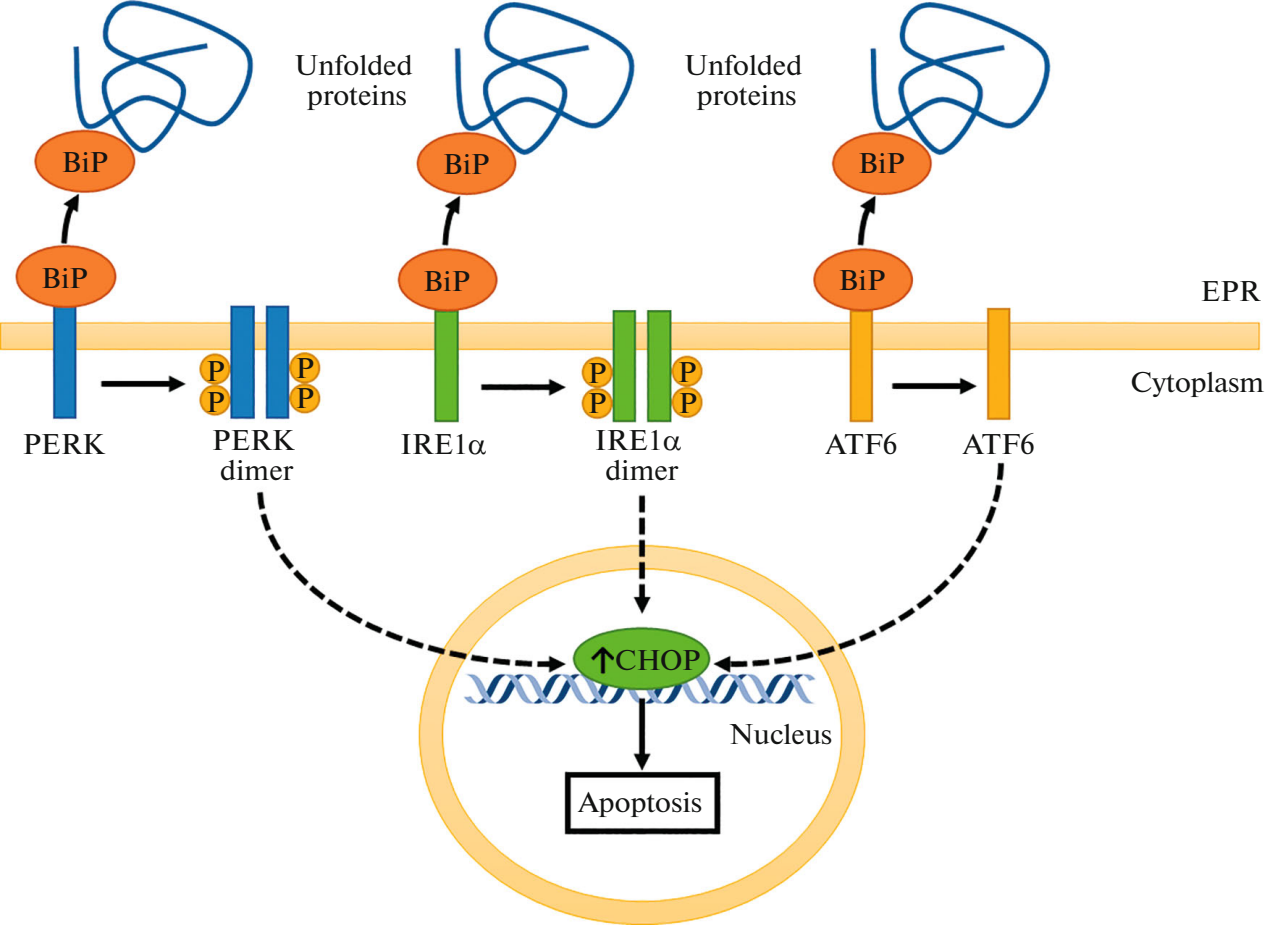
Signaling pathway of the unfolded protein response during simulation of hypoxia with CoCl_2_. Unfolded proteins in the endoplasmic reticulum (ER) bind to the BiP protein, which releases the PERK, IRE1α, and ATF6 proteins. All three branches of this pathway are aimed at compensating for endoplasmic stress; however, upon failure of adaptation, they increase the expression of the proapoptotic transcription factor CHOP.

Analysis of the genes whose expression significantly changed uniquely for the effect of HD revealed a decrease in oxidative phosphorylation, which is a normal response of trophoblast cells during the transition from aerobic to anaerobic metabolism during hypoxia [19].

It should be specially noted that, according to the analysis of signaling pathways in BeWo b30 cells, the exposure to HD suppresses the mTORC1 signaling pathway, whereas the exposure to CoCl_2_ causes its activation. It is known that hypoxia suppresses the mTORC1 signaling pathway [20]. Under the influence of CoCl_2_, the expression of the *RHEB* gene increased 1.9 times and the expression of the *TSC2* gene decreased 2.5 times. However, the expression of these genes under the influence of HD did not change significantly, which may explain the activation of the mTORC1 pathway under the influence of CoCl_2_, in contrast to HD.

Figure 3 shows a simplified scheme of one of the control mechanisms of the mTORC1 pathway and shows the revealed effect of CoCl_2_ on the *TSC2* and *RHEB* genes.

**Fig. 3.**
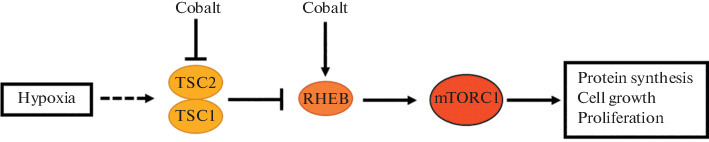
mTORC1 signaling pathway during hypoxia. Hypoxia increases the activity of the TSC1/TSC2 complex, which inactivates the RHEB protein; this leads to the suppression of mTORC1 activity. Under the influence of CoCl_2_, the expression of TSC2 is decreased and the expression of RHEB is increased, which may explain the activation of the mTORC1 pathway.

Thus, the comparison of two chemical models of hypoxia—exposure to HD and CoCl_2_—revealed activation of the hypoxia signaling pathway and the epithelial–mesenchymal transition in the trophoblast, as well as suppression of cell proliferation under both exposures. However, the exposure to cobalt activated the genes that are involved in the apoptosis signaling pathway and more significantly increased the activity of caspases 3 and 7 in comparison with the control conditions and the exposure to HD. CoCl_2_ activated the pathway of the unfolded protein response and mTORC1, whereas HD suppressed the mTORC1 pathway. All this indicates that the model of chemical hypoxia induced by HD reflects the effect of moderate hypoxia on trophoblast cells, whereas CoCl_2_ has a damaging effect accompanied by pronounced apoptosis. From a clinical point of view, the differences in the activation of signaling pathways in moderate and severe hypoxia may indicate the necessity for different approaches to the treatment of these conditions. Apoptosis and impaired invasion of the trophoblast into the mother’s vessels in severe hypoxia may be a prerequisite for the development of a severe complication of pregnancy, preeclampsia [1]. HIF-1α activation by HD can trigger an antihypoxic response in cells, preparing the cell to respond to severe hypoxia, which has a neuroprotective effect in stroke and is used in the treatment of anemia associated with renal failure [9]. The results obtained create prerequisites for the development of approaches to the prevention of the development of hypoxic complications of pregnancy.

## Abbreviations

HIF: hypoxia-inducible factor
PHD: prolyl hydroxylases
pVHL: von Hippel Lindau protein
ROS: reactive oxygen species
HD: hydroxyquinoline derivative
EPR: endoplasmic reticulum
